# Paraurachal paraganglioma

**DOI:** 10.1002/iju5.12488

**Published:** 2022-05-31

**Authors:** Masafumi Tsuruta, Takayuki Goto, Jin Kono, Yuki Kita, Kimihiko Masui, Takeshi Sano, Masakazu Fujimoto, Atsuro Sawada, Shusuke Akamatsu, Takashi Kobayashi

**Affiliations:** ^1^ Department of Urology Kyoto University Hospital Kyoto Japan; ^2^ Department of Diagnostic Pathology Kyoto University Hospital Kyoto Japan

**Keywords:** chromaffin cells, paraganglioma, urachus

## Abstract

**Introduction:**

Paragangliomas (PGLs) are frequently reported around the abdominal aorta; however, are extremely rare near the urachus.

**Case presentation:**

A 78‐year‐old woman was referred to the urology department of our hospital for further examination and treatment of a 1.2‐cm tumor in the lower abdominal wall, a tumor excision was then performed. On immunohistochemical staining, the tumor and supporting cells were positive for chromogranin A and the S 100 protein, respectively, and were diagnosed as PGL. The PGL was thought to be derived from chromaffin cells that migrated to the wall of the urachus during embryonic life and remained even after the wall regressed.

**Conclusion:**

We report a case of PGL near the urachus that can be explained by the distribution of the sympathetic network around the midline of the lower abdominal wall during embryonic development. Therefore, PGL should be considered in the differential diagnosis of periurachal tumors.

**Abbreviations & Acronyms**
CT: Computed tomogrphy
PGL: Paragangliomas
SDHB: Succinate Dehydrogenase B

**Keynote message**

We report a case of PGL that arose around the urachus and was thought to be derived from the chromaffin cells that migrated to the wall of the urachus during embryonic development and remained there even after the involution of the urachus.

## Introduction

Paragangliomas (PGLs) are rare neuroendocrine tumors that produce catecholamines. They are frequently reported around the abdominal aorta.^1^ In this study, we report a rare case of PGL around the urachus.

## Case presentation

**Fig. 1:**
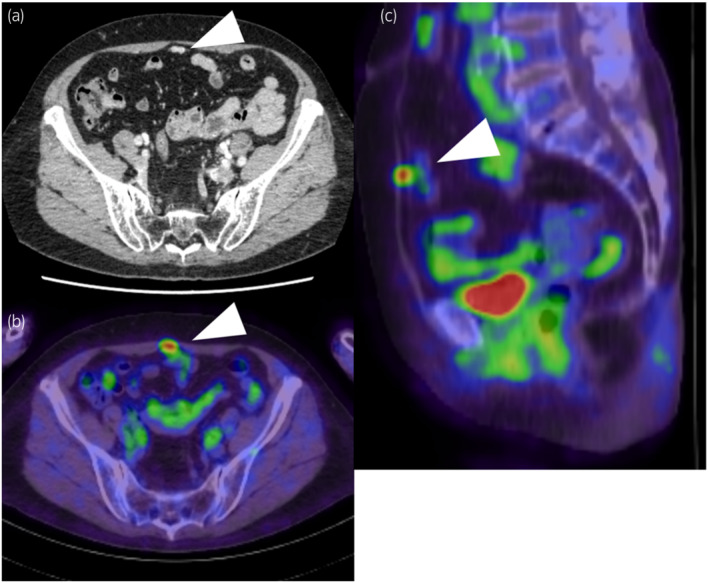
Images of the lower abdominal tumor (arrowhead). (a) A 1.2‐cm tumor was found in the midline of the lower abdominal wall on an axial view of the computed tomography (CT). (b), (c) Axial and sagittal view of fluorodeoxyglucose positron emission tomography‐computed tomography (FDG‐PET/CT) showed intense FDG uptake (maximum standardized uptake value, SUV_max_, 4.51).

A 78‐year‐old woman was referred to our hospital for treatment of duodenal papillary cancer. Fluorodeoxyglucose positron emission tomography‐computed tomography (FDG‐PET/CT) showed a 1.2‐cm tumor in the midline of the lower abdominal wall (Fig. 1), and she was referred to the urology department with a suspected urachal carcinoma. CT showed no signs of urachus remnant.

To rule out peritoneal dissemination of duodenal papilla cancer, excision of the lower abdominal wall tumor was performed prior to the operation for duodenal papilla cancer, and peritoneal dissemination was evaluated. We performed a midline incision above the umbilicus in accordance with the dermatome of the open pancreaticoduodenectomy. The tumor was located in the retroperitoneum near the urachus and was not exposed to the peritoneum. Because no malignant findings were observed in the intraoperative rapid pathological examination, peritoneal dissemination was considered negative, and pancreaticoduodenectomy was performed on the same day. Tumor manipulation during surgery increased the systolic blood pressure from 90 to 180 mmHg, and it decreased after tumor extraction that required temporary use of a vasopressor.

**Fig. 2:**
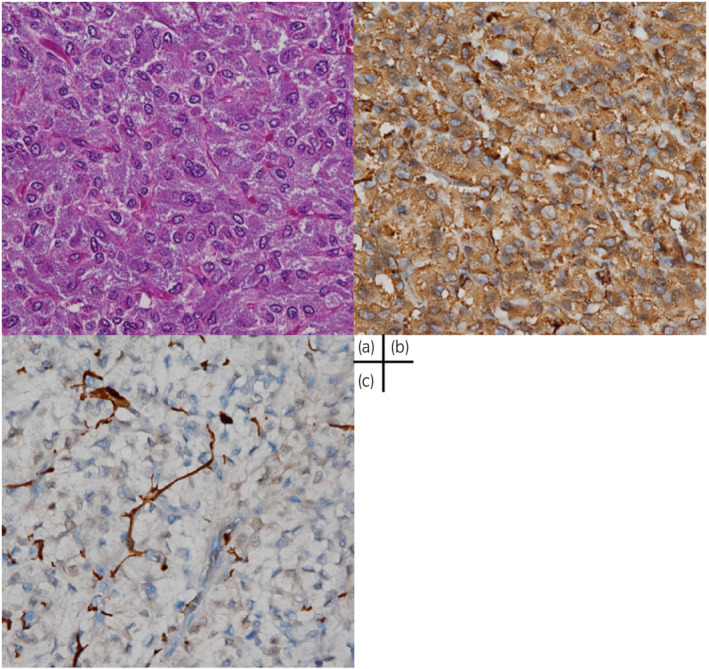
On histopathological examination, the tumor had basophilic granular cytoplasm, surrounded by fibrovascular stroma (hematoxylin–eosin, 400×) (a). On immunohistochemical staining, the tumor cells are positive for chromogranin A (400×) (b), and supporting cells are positive for S 100 protein (400×) (c).

On histopathological examination, the tumor had a basophilic granular cytoplasm surrounded by a fibrovascular stroma. On immunohistochemical staining, the tumor cells were positive for chromogranin A and the supporting cells were positive for the S 100 protein, and were diagnosed as PGL (Fig. 2). Remnants of the urachus were not identified, and only the fibrous cord‐like structure which seemed to be the scar of the urachus with involution was recognized. The Grading System for Adrenal Pheochromocytoma and Paraganglioma (GAPP) was given a score of 2 for the noradrenaline type and 1 for the non‐noradrenaline type. Either way, it is a Well differentiated type. Succinate Dehydrogenase B (SDHB) immunostaining was positive, indicating that *SDHA*, *SDHB*, *SDHC*, or *SDHD* gene mutations were negative. Pathological examination of the duodenal papilla cancer revealed well differentiated adenocarcinoma with papillary and tubular growth in the mucosa of the papillary bile duct, and the pathological stage was pT1bN0.

According to the medical history obtained postoperatively, she had episodes of paroxysmal hypertension in her late 40s with a systolic blood pressure of 180–200 mmHg, accompanied by palpitations, headaches, cold sweats, and paleness. However, after the surgery, the patient did not experience similar attacks. There was no association between paroxysmal attacks and urination/urination, probably due to the distance from the bladder. She had been prescribed amlodipine and atenolol by her family doctor. After the surgery, she also improved hypertension and no longer needed two kinds of the antihypertensive drugs. Her father had refractory hypertension, and her daughter had a hypertensive episode similar to the patient's. Her daughter underwent catecholamine examination in urine and blood and 123 I metaiodobenzylguanidine (MIBG) scintigraphy, and no abnormal findings were observed.

Postoperatively, she underwent 123 I MIBG scintigraphy and examinations to check for catecholamines in the blood and urine. The scintigraphy report revealed no significant integration, and the catecholamine test result was within the normal range. She was offered a genetic test but did not take it. Because the GAPP score was low, the follow‐up was requested to the endocrine medicine and the catecholamine inspection is periodically enforced. The imaging examination was periodically carried out in the recurrence follow‐up of the duodenal papilla cancer. There was no apparent recurrence 2 years after the operation.

## Discussion

Chromaffin cells migrate from the neural crest at 5 weeks of gestation and contribute to the formation of the adrenal medulla.^2^ On the other hand, ectopic chromaffin cells often produce PGLs around the aorta, consistent with the distribution of paraganglia.^1^ Abdominal wall involvement is rare, reported only in paravesical PGL.^3^ Although PGL as a bladder submucosal tumor is well known in urology, this is the first report of PGL around the urachus.^4^

In fetal life, the muscular layer continues from the bladder wall to the allantoic duct; however, the muscular layer of the allantoic duct disappears by 15–20 weeks of gestation.^5^ Therefore, it is reasonable to think that the cells derived from the neural crest may extend further around the urachus as they migrate to the bladder wall. There have been several reports of periurachal neuroblastoma, and these reports support cell migration from the neural crest to the periurachal region because neuroblastomas arise from neural crest.^6^, ^7^ Thus, we consider that chromaffin cells migrated from the neural crest through the bladder wall to the wall of the urachus during embryonic development and remained there even after involution of the allantoic muscular layer, giving rise to PGL in the present case.

Another possible hypothesis is that aberrant chromaffin cells accidentally originated from the peripheral nerve cells in the abdominal wall. However, chromaffin cells are said to arise from Schwann cell precursors that change to immature Schwann cells during embryonic development.^8^, ^9^ Therefore, it is unlikely that chromaffin cells develop abruptly from mature peripheral nerves. Moreover, there has been no report of PGL arising far from the area of chromaffin cell migration.

The present case provides an implication of chromaffin cell distribution during development. Clinically, this case suggests that PGL should be considered as a differential diagnosis for tumors around the urachus.

## Author contributions

Masafumi Tsuruta: Conceptualization; data curation; writing – original draft. Takayuki Goto: Conceptualization; writing – review and editing. Jin Kono: Writing – review and editing. Yuki Kita: Writing – review and editing. Kimihiko Masui: Writing – review and editing. Takeshi Sano: Writing – review and editing. Masakazu Fujimoto: Data curation; writing – review and editing. Atsuro Sawada: Writing – review and editing. Shusuke Akamatsu: Writing – review and editing. Takashi Kobayashi: Conceptualization; supervision; writing – review and editing.

## Conflict of interest

The authors declare no conflict of interest.

## Compliance with ethical standards

All procedures performed in this study involving human participants were in accordance with the ethical standards of the institutional and/or national research committee and with the 1964 Helsinki declaration and its later amendments or comparable ethical standards.

## Approval of the research protocol by an institutional reviewer board

The protocol for this research project has been approved by our institutional reviewer board Approval No. R1531.

## Informed Consent

Not applicable.

## Registry and Registration No. of the study/trial

Not applicable.
